# Home-Made Soap

**Published:** 1890-10

**Authors:** 


### HOME-MADE SOAP.

I have found a way in which I can make soap while waiting for the kettle to boil for supper. It is very easy. Get of a druggist or grocer, a pound box of the pulverized lye now sold so cheaply, and in such convenient shape. It will cost you fifteen cents. It comes in a neat can, which can be opened with any penknife. Dissolve this lye in three pints of water. The lye heats the water and you must wait till this heat passes off before making your soap. Melt your grease, and strain through a cheese-cloth, and weigh five and a half pounds. As soon as this melted grease is cool enough to bear your hand in, pour grease and lye together, and stir thoroughly a few minutes, and you will see it thicken. Now pour it into a box or dripping pan lined with greased paper, and let it stand in a warm place for twenty-four hours, then cut into bars. It will be ready for immediate use, will keep growing better, is clean and thoroughly satisfactory for dish washing and the laundry, makes a good suds, and is economical, having cost you only fifteen cents, the price of your lye, as the grease was saved at odd times. It can be made without fire, as you see it does not have to be boiled, or either have boiling water added. Our laundress uses it and says, "It is good," and she is apt to be critical.—*Good Housekeeping*.

